# Posttranscriptional and transcriptional regulation of endothelial nitric-oxide synthase during hypoxia: the role of microRNAs

**DOI:** 10.1186/s11658-016-0017-x

**Published:** 2016-09-06

**Authors:** Leszek Kalinowski, Anna Janaszak-Jasiecka, Anna Siekierzycka, Sylwia Bartoszewska, Marcin Woźniak, Dawid Lejnowski, James F. Collawn, Rafal Bartoszewski

**Affiliations:** 1 Department of Medical Laboratory Diagnostics and Central Bank of Frozen Tissues & Genetic Specimens, Medical University of Gdansk, Debinki 7, 80-211 Gdansk, Poland; 2 Department of Biology and Pharmaceutical Botany, Medical University of Gdansk, Hallera 107, 80-416 Gdansk, Poland; 3 Department of Inorganic Chemistry, Medical University of Gdansk, Gdansk, Poland; 4 Department of Cell Biology, Developmental, and Integrative, University of Alabama at Birmingham, Birmingham, USA

**Keywords:** eNOS, ER stress, Hypoxia, miRNA, Nitric oxide, NO bioavailability, *NOS3*, *sONE*

## Abstract

Understanding the cellular pathways that regulate endothelial nitric oxide (eNOS, *NOS3*) expression and consequently nitric oxide (NO) bioavailability during hypoxia is a necessary aspect in the development of novel treatments for cardiovascular disorders. eNOS expression and eNOS-dependent NO cellular signaling during hypoxia promote an equilibrium of transcriptional and posttranscriptional molecular mechanisms that belong to both proapoptotic and survival pathways. Furthermore, NO bioavailability results not only from eNOS levels, but also relies on the presence of eNOS substrate and cofactors, the phosphorylation status of eNOS, and the presence of reactive oxygen species (ROS) that can inactivate eNOS. Since both *NOS3* levels and these signaling pathways can also be a subject of posttranscriptional modulation by microRNAs (miRNAs), this class of short noncoding RNAs contribute another level of regulation for NO bioavailability. As miRNA antagomirs or specific target protectors could be used in therapeutic approaches to regulate NO levels, either by changing *NOS3* mRNA stability or through factors governing eNOS activity, it is critical to understand their role in governing eNOS activity during hypoxa. In contrast to a large number of miRNAs reported to the change eNOS expression during hypoxia, only a few miRNAs modulate eNOS activity. Furthermore, impaired miRNA biogenesis leads to *NOS3* mRNA stabilization under hypoxia. Here we discuss the recent studies that define miRNAs’ role in maintaining endothelial NO bioavailability emphasizing those miRNAs that directly modulate *NOS3* expression or eNOS activity.

## Introduction

The signaling molecule nitric oxide (NO) produced in endothelium by nitric oxide synthase 3 (eNOS, encoded by *NOS3* gene) plays a pivotal role in the maintenance of homeostasis in the blood vessel wall [1–3]. eNOS produces nitric oxide through conversion of L-arginine and oxygen into L-citrulline and NO [4]. Rapid NO diffusion to vascular smooth muscle cells leads to guanosine 3,5-cyclic monophosphate formation and consequently vasodilation [4]. Hence, impaired activity of eNOS and the loss of NO bioavailability are associated with endothelial cell dysfunction that is an independent risk factor for cardiovascular diseases [1, 2]. Endothelium dysfunction due to the reduction of NO bioavailability in the vessel wall is one of the earliest manifestations of atherosclerosis and hypertension [5]. Although eNOS targeting is an attractive approach in terms of preventing and treating atherosclerosis and other cardiovascular disorders, the phenomenon of eNOS uncoupling hampers the attempts to assess whether eNOS-derived NO serves to protect vessels from the progression of atherosclerosis. eNOS must be regarded as both an NO and an O_2_^-^-producing enzyme, and therefore, eNOS may have dual effect on vascular function, depending on its functional state [6–11]. Hence, future therapeutic approaches should rely on the physiologically relevant restoration of endothelial NO homeostasis via modulation of eNOS activity rather than just overexpression of this enzyme.

In endothelial cells, *NOS3* is constitutively expressed. *NOS3* message, however, is strongly susceptible to upregulation by many factors including: ROS [12]; laminar and oscillatory shear stress [13, 14], and cell growth [15]. The eNOS activity is regulated via two mechanisms, calcium/calmodulin binding or phosphorylation by serine/threonine-specific kinase (Akt) [16]. However, different physiological and pathological conditions have been shown to affect *NOS3* expression via both the transcriptional [17–19] and post-transcriptional mechanisms [20–23]. Significantly, hypoxia/ischemia is a major cellular stress that has a profound impact on endothelial cell biology, including cardiovascular pathologies. Thus, understanding the molecular mechanisms regulating *NOS3* gene expression under low oxygen tension is a high-impact priority. Importantly, prolonged hypoxia and ischemia lower endothelial *NOS3* expression, leading to a loss of NO bioavailability [23, 24]. In these instances, a major contributing factor to downregulation of *NOS3* expression appears to be a reduction in the stability of *NOS3* mRNA. This decrease in *NOS3* mRNA abundance is attributed, at least in part, to the destabilization of *NOS3* mRNAs by a natural overlapping antisense transcript to *NOS3* called *sONE* [23]. However during hypoxia, specific miRNAs may reduce endothelial *NOS3* levels and consequently modulate the bioavailability of NO [25]. Furthermore, hypoxia often disturbs endoplasmic reticulum (ER) homeostasis, leading to ER stress response activation [26]. Hence, besides hypoxia-related transcription factors and miRNAs, the transcriptional and post-transcriptional mediators (miRNAs) of the ER stress pathway might also influence endothelial NO bioavailability.

## Cardiovascular disorders and NO bioavailability

The endothelium plays a crucial role in regulating vascular function. Although serving as an extremely active endocrine and paracrine organ that produces a large variety of molecules participating in complex biochemical processes, the simple product generated by eNOS - NO - seems to be a key molecule required for the maintenance of vascular homeostasis [1–3, 7, 8]. For example, NO produced by eNOS causes vasodilation. Thus, *NOS3* knockout mice are hypertensive [27], whereas *NOS3* transgenic mice exhibit hypotension [28]. In addition, NO reduces the activation and aggregation of platelets, attenuates adhesion of leukocytes to the endothelium, reduces the permeability of the endothelium, and inhibits proliferation and migration of vascular smooth muscle cells [29, 30]. Impaired activity of eNOS and the loss of NO bioavailability are associated with endothelial cell dysfunction that is an independent risk factor for cardiovascular diseases [1, 2]. A number of models of endothelial dysfunction in experimental animals together with clinical data provided evidence that NO bioavailability is reduced by increased production of reactive oxygen species (ROS) in the vessel wall. Of ROS, superoxide (O_2_^–^) is the key molecule as many other ROS are formed secondary to the reactions involving O_2_^–^. Because O_2_^–^ and NO are both radicals and contain unpaired electrons in their outer orbitals, they undergo an extremely rapid, diffusion limited radical-radical reaction, leading to the formation of peroxynitrite (ONOO^–^), a much stronger oxidant than O_2_^–^ itself. There is growing evidence that an imbalance between production NO and O_2_^-^ within the endothelium can contribute to the onset of a variety of cardiovascular disease states such as atherosclerosis, thrombosis, hypertension, diabetes mellitus, heart failure, post-angioplasty restenosis, cerebral vasospasm and delayed wound healing [1, 2, 7, 8]. Many of these disorders are associated with hypoxia or ischemia in different organs and this leads to a decrease in oxygen and nutrient delivery to the tissues. Importantly, hypoxia and ischemia lower endothelial NOS3 expression, leading to loss of NO bioavailability.

## eNOS uncoupling

Because O_2_^-^ avidly scavenges NO, a reduction of bioactive NO may occur despite an increased NO generation. Among several enzymatic systems that are capable of producing O_2_^-^, eNOS itself is a significant source of O_2_^-^ in the vessel wall [1, 7, 9, 10, 31]. In the absence of a substrate, L-arginine, or a cofactor such as tetrahydrobiopterin (BH_4_), eNOS synthesizes O_2_^-^ in preference to NO. In the process of eNOS dysfunction called enzyme “uncoupling”, the electron flow through the eNOS enzyme is then diverted to molecular oxygen rather than to L-arginine, which facilitates the production of O_2_^-^ rather than NO [7–9, 11]. Furthermore, during pathological conditions, eNOS mediates formation of peroxynitrite (ONOO^-^) [32–34]. Hence, the extent of eNOS uncoupling is dependent on ONOO^-^ produced initially in the reaction between NO produced by eNOS and O_2_^-^ generated by both NAD(P)H oxidase and eNOS [35, 36]. It has also been suggested that both the zinc-thiolate center of eNOS and BH_4_ are probable targets of oxidation by ONOO^-^ [37]. This in turn can lead to dissociation of eNOS dimers to monomers with the subsequent release of zinc cations [38]. Furthermore, cellular studies have shown that phosphorylation of eNOS at specific amino acids (Ser-1177, Thr-495) can regulate enzyme-mediated production of both NO and O_2_^-^ [39]. eNOS uncoupling partially occurs even in normal endothelium and may explain the predisposition of some individuals to endothelial dysfunction and cardiovascular complications. Although eNOS targeting is an attractive approach in terms of preventing and treating atherosclerosis and other cardiovascular disorders, the phenomenon of eNOS uncoupling hampers the attempts to assess whether eNOS-derived NO serves to protect vessels from the progression of atherosclerosis. eNOS must be regarded as both an NO and an O_2_^-^-producing enzyme, and therefore eNOS may have dual effect on vascular function, depending on its functional state [6–11].

## Regulation of eNOS expression during hypoxia

**Fig. 1:**
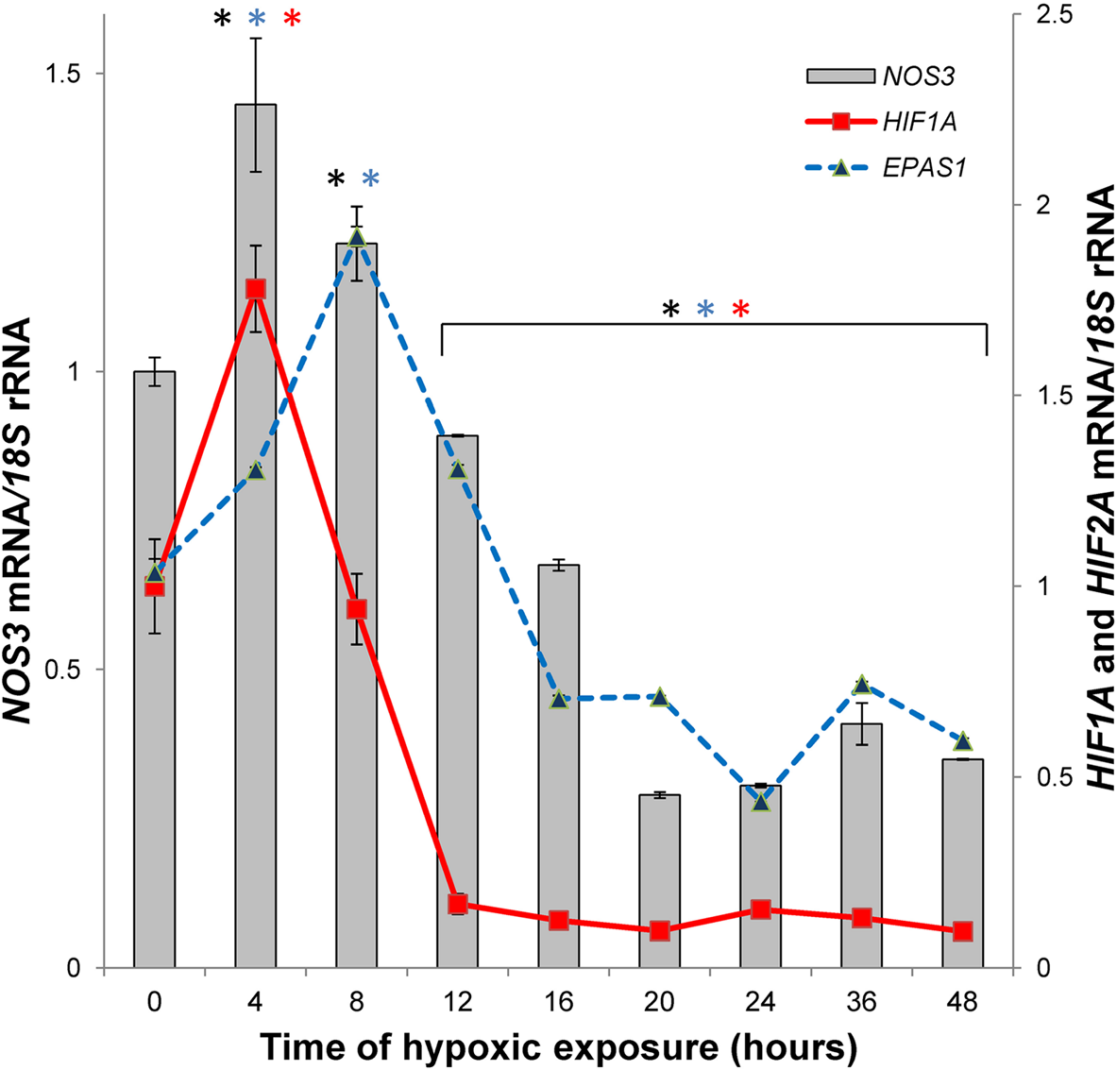
Hypoxia induces dynamic changes in the mRNA expression profiles of the *NOS3*, *HIF1A* and *HIF2A (EPAS1)* in human human umbilical vein endothelial cells (HUVECs). The mRNA levels during hypoxia were monitored in qRT-PCR experiments as described in [49, 50]. The results from 2 independent experiments (*n* = 8) are plotted normalized to 18S rRNA levels and expressed as a fold-change compared to the normoxic control. The hypoxia had no impact on HUVECs viability as monitored with Real Time xCelligence as described in [106]. Error bars represent standard deviations. Significant changes (*p* < 0.05) are marked with an “*”

The molecular basis of hypoxic vasodilation is not fully understood [40] and the various effects of hypoxia on eNOS in endothelial cells have been described previously [24]. Under normoxia, the *NOS3* mRNA is highly stable in human endothelium [41]. However, eNOS activity and *NOS3* expression levels are increased by hypoxia in some reports [42–46], whereas in others they either decreased or not affected [24, 47, 48]. The discrepancies between these reports result from the duration of exposure to hypoxia as well as from the different endothelial models used. Importantly, the prolonged hypoxia usually causes a decrease in *NOS3* expression, while short-term oxygen depletion leads to *NOS3* mRNA accumulation in HUVECs (Fig. 1). The early hypoxic induction of *NOS3* expression was shown to rely on hypoxia-responsive elements (HRE) at position _5375 to _5366 (relative to the transcription start site) [19]. *NOS3* luciferase reporter studies illustrated that both of these HREs were functional for *NOS3* promoter activity induction by hypoxia and by HIF-2 overexpression [19]. Hence, during early hypoxia, HIFs and especially HIF-2 have been shown to induce *NOS3* expression. Indeed, we previously demonstrated that HIF-1 is induced and accumulates at the early stages of hypoxia in primary endothelial cells and this positively correlates with the *NOS3* mRNA expression profile [49, 50]. However, the HIF-2 expression increases at later stages of hypoxia [50] correlated with *NOS3* mRNA levels, presumably stabilizing *NOS3* mRNA. Thus, further studies are required in order to better understand the role HIFs in *NOS3* expression modulation in response to hypoxia.

Under prolonged hypoxia, the reduction in stability of the mature *NOS3* mRNA is a major contributing factor to down-regulation of *NOS3* expression [25]. Notably, numerous studies have shown that hypoxia significantly down-regulates *NOS3* mRNA expression in endothelial cells [23, 24]. Furthermore, experimental models of proliferation/injury [51], tumor necrosis factor alpha treatment [52], and exposure to lipopolysaccharide [53] all decrease *NOS3* steady-state mRNA expression in endothelial cells. To date, numerous mechanisms responsible for *NOS3* mRNA hypoxic reduction have been proposed. The initial studies postulated that *NOS3* mRNA destabilization during hypoxia is inhibited by a Rho kinase inhibitor [54]. We have provided clear evidence that Rho GTPase proteins negatively regulates eNOS expression and HMG-CoA reductase inhibitors (statins) upregulate eNOS expression by blocking Rho geranylgeranylation, which is necessary for its membrane-associated activity [55, 56]. However, recent studies attributed the hypoxic decrease in *NOS3* mRNA abundance, at least in part, to the destabilization of *NOS3* mRNAs by *sONE* (also known as *ATG9B*, *NOS3AS*, and *APG9L2*) [57]. The *NOS3* and *sONE* genes are arranged in a tail-to-tail orientation on human chromosome 7q36, and the transcripts for the two genes are complementary for a total of 662 nucleotides (including exon/exon overlap). During normoxia, the *sONE* transcript is expressed at very low levels in endothelial cells, whereas *NOS3* is highly abundant [25]. Importantly, long-term hypoxia, which downregulates *NOS3* mRNA and protein expression, significantly upregulates steady-state levels of *sONE* RNA [23]. Importantly, basal expression of *NOS3* mRNA is stabilized by the formation of ribonucleoprotein RNP complexes on 3′-UTR *cis* elements. Recent studies identified heterogenous nuclear RNP (hnRNP) E1, a ubiquitous, multifunctional RNA-binding protein [25], as a major component of these eNOS-stabilizing RNP complexes [25]. Significantly, the stabilization of *NOS3* mRNA by hnRNP E1 complexes constitutes a protective mechanism against the posttranscriptional inhibitory effects of the *NOS3* antisense transcript *sONE* and miRNAs (miR-765) during normoxic conditions [25]. In normoxia, the hnRNP E1 complexes at *NOS3* 3′-UTR prevent *sONE* and miRNA binding. However, hypoxia disrupts hnRNP E1/*NOS3* 3′-UTR interactions via increased Akt-mediated serine phosphorylation and nuclear localization of hnRNP E1, while hnRNP E1 levels remain constant [25]. Hence, under hypoxic conditions, removal of hnRNP-mediated protection of *NOS3* mRNA makes this transcript susceptible to *sONE* and miRNAs-related down-regulation.

All of the studies discussed above imply that hypoxic *NOS3 mRNA* levels are at equilibrium that result from the simultaneous interplay between HIF transcriptional induction of *NOS3* promoter and destabilization of *NOS3* mRNA. Furthermore, the factors that modulate HIF signaling or *sONE* levels, such as miRNAs, could consequently influence *NOS3* mRNA expression, and subsequently eNOS protein levels, and thus NO bioavailability.

## Hypoxia-related ER stress activation

**Fig. 2:**
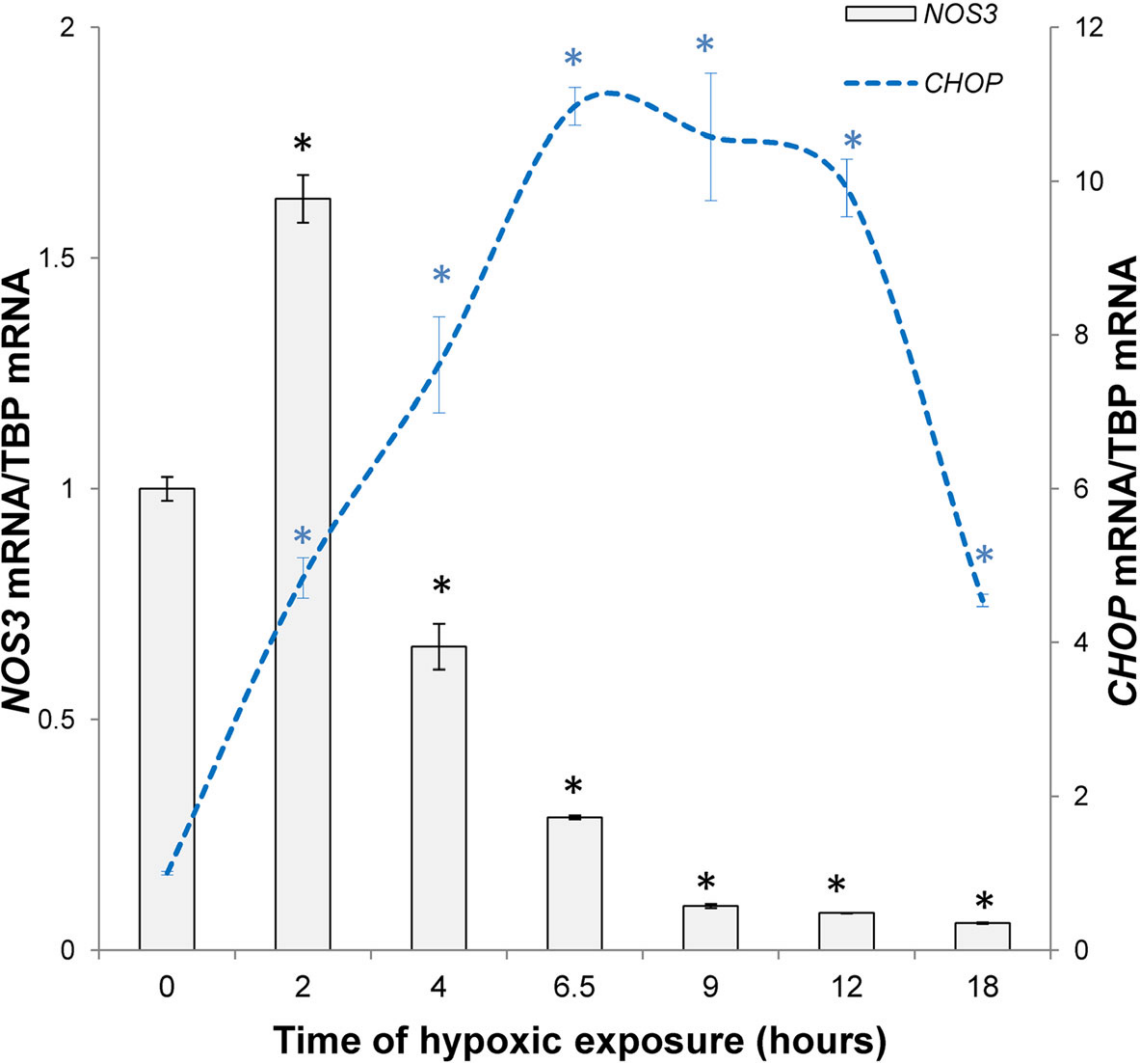
The ER stress induced dynamic changes in the mRNA of the *NOS3* that are negatively correlated with CHOP expression in human umbilical vein endothelial cells (HUVECs). The ER stress was induced with 100 μM Calpain Inhibitor I (ALLN). mRNA levels were monitored in qRT-PCR experiments as described in [106, 107]. The results from 2 independent experiments (*n* = 8) are plotted normalized to TBP mRNA levels and expressed as a fold-change over the untreated control. Error bars represent standard deviations. Significant changes (*p* < 0.05) are marked with an “*”

Recent studies also provided compiling evidence that disturbed ER function plays a crucial role in a number of hypoxia-triggered endothelial patholophysiological processes leading to cardiovascular disorders such as atherosclerosis, ischemic cardiac and peripheral vascular diseases, and neovascularization [58–60]. Hypoxia triggers endothelial ER stress and apoptosis, and induces very low density lipoprotein (VLDL) receptor (VLDLr) expression through HIF-1 transcriptional activity [61]. Since eNOS protein biogenesis occurs in the ER, hypoxia-related ER stress activation could also modulate *NOS3* expression at both the transcriptional and posttranscriptional levels [62]. Importantly, chemical ER stress induction was shown to decrease *NOS3* mRNA (Fig. 2) via a negative transcriptional effect on the proapoptotic transcription factor C/EBP homologous protein (CHOP, also known as growth arrest and DNA damage gene 153) [63]. CHOP is expressed at low levels during physiological conditions, but is dramatically up-regulated in response to ER stress [64]. CHOP is a major mediator of apoptosis, and has recently been shown to regulate angiogenesis [65]. During the early adaptive stages of ER stress, we observed *NOS3* mRNA induction (Fig. 2), suggesting the involvement of another mechanism. In line with this, it was reported that overexpression of active form of proadaptive ER stress transcription factor (XBP1s) upregulates both *NOS3* mRNA and protein levels and leads to an increase in NO production [66]. Overexpression of XBP1s increases Akt phosphorylation [67], whereas the phosphorylation of *NOS3* by Akt represents a major Ca^2+-^independent regulatory mechanism for the activation of eNOS [68].

Hence, the hypoxic activation of ER stress additionally complicates the mechanism for maintaining *NOS3* mRNA levels and contributes at least two other important transcription factors oppositely modulating *NOS3* mRNA abundance - survival XBP1 and proapoptotic CHOP.

## microRNAs - Regulation of NO production

miRNAs are short, single-stranded RNA molecules approximately 22 nucleotides in length that that play key roles in the posttranscriptional regulation of gene expression. As a component of miRNA-induced silencing complex (RISC), miRNAs initiate mRNA decay and thus reduce protein output. The base-pairing interactions between nucleotides 2 and 8 of the miRNA (the seed region) and complementary nucleotides in the 3′-untranslated region (3′-UTR) of mRNA are responsible for the miRNA specificity [69]. Recent studies, however, indicate that miRNAs may play critical roles during hypoxia [70] and ER stress [71].

Despite the fact that inhibiting miRNAs function (through DICER silencing [25]) prevents *NOS3* down-regulation during hypoxia, only four miRNAs that directly affect *NOS3* expression have been identified that include *miR-214* [72], *miR-155* [73], *miR-24* [74] and *miR-765* [25].

*miR-214* was reported to be induced in myocardial hypoxia as a indirect result of HIF-1 activity [75]. In Human Umbilical Vein Endothelial Cells (HUVECs), downregulation of *miR-214* resulted in increased *NOS3* expression (in normoxia) and miRNA binding at *NOS3* 3′UTR was verified [75]. Hence, the hypoxic induction of *miR-214* could be another factor destabilizing *NOS3* message.

Direct binding of *miR-24* to *NOS3* mRNA was confirmed in HUVECs and resulted in lowering eNOS protein levels in normoxia [74]. Although independent studies reported *miR-24* induction during hypoxia in human neuroblastoma cells [76], the expression profile of this miRNA during hypoxia in human endothelium requires further study. Recently, it was also proposed that XBP1-related down-regulation of some miRNAs including *miR-24*, *miR-125* and *miR-214* may lead to *NOS3* mRNA stabilization [66]. However, the role of ER stress and especially ER stress-specific miRNAs in modulating *NOS3* mRNA levels requires further study.

*miR-155,* that is induced during hypoxia in intestinal epithelial cells [77], was also reported to directly bind to *NOS3* 3′UTR and reduce eNOS levels in human endothelium [73]. *miR-765*, which is an abundant species (within the top 20 % of all detected microRNAs) in HUVEC [78], binds to *NOS3* mRNA at 3′ UTR region that is stabilized in normoxia by hnRNP [25]. During hypoxia, however, hnRNP dissociates from *NOS3* mRNA sensitizing this transcript to miR-765-dependent degradation [25].

*miR-155, miR-214*, and *miR-24* were reported to destabilize *NOS3* mRNA in normoxia via the hnRNP-independent pathway. Therefore changes in these miRNAs expression profiles modulate *NOS3* mRNA levels during hypoxia. On the other hand, *miR-765* has no effect on *NOS3* message in normoxia, while the effects of *miR-765* on *NOS3* mRNA stability are hnRNP-dependent during hypoxia. Hence, we have example of different mechanisms of miRNA-dependent *NOS3* mRNA regulation during hypoxia. The hnRNP-independent pathway allows dynamic and bidirectional modulation of *NOS3* mRNA stability up on induction or repression of miRNAs expression during hypoxia. The hnRNP- dependent pathway (also utilized by *sONE*) allows rather constitutive downregulation of *NOS3* mRNA during hypoxia and does not necessarily require respectful change in miRNAs levels (especially if such a microRNAs are abundant).

Importantly HIF-1, the main transcriptional mediator of cellular responses to hypoxia, promotes the expression of several hypoxamiRs including *miR-155* in intestinal epithelial cells [77]. Furthermore, *miR-155* has been shown to negatively regulate *HIF1A* message levels, establishing a negative regulatory feedback loop during hypoxia [77]. Interestingly, the cytokine TNF-α, known to dramatically destabilize *NOS3* mRNA [79], is also responsible for induction of *miR-155* [80]. Thus, *miR-155* provides a sensitive link between transcriptional and posttranscriptional mechanisms that regulate *NOS3* expression during hypoxia through its actions on *HIF-1A* and *NOS3* mRNA levels.

HIF-1 and HIF-2, therefore, are the perfect candidates for controlling *NOS3* expression and *NOS3*-related cellular signaling during hypoxia. Interestingly, no miRNA has been shown to control *sONE* nor *HIF-2A* levels in human endothelium.

Recently, the hypoxamiR *miR-101* was shown to indirectly enhance the interaction between HIF-1α and Hsp90 that resulted in both increased VEGF expression and eNOS activity [81]. Both HIF-1 and HIF-2 induce proangiogenic vascular endothelial growth factor (VEGF) expression [82]. VEGF subsequently leads to activation of Akt and thus increased NO levels [83, 84]. Furthermore, hypoxia upregulates heat shock protein 90 (Hsp90) expression in endothelial cells [85].^.^ Hsp90 binds to the α subunits of HIF-1, protecting this protein from oxygen-independent degradation and sustaining HIF-1 transcriptional activity [86]. Importantly Hsp90 also increases eNOS activity since it is required for the interaction of eNOS with Akt [87], and Hsp90 binding to eNOS leads to increased affinity for calmodulin binding as well [88].

The other hypoxamiRs that modulate Akt phosphorylation during hypoxia could potentially indirectly affect NO levels as well. To date, numerous hypoxamiRs were shown to regulate Act activity indirectly. *miR-21*, *miR-26*, *miR-221/222*, and *miR-486* bind to phosphatase and tensin homolog deleted on chromosome 10 (PTEN) and, thus activate Akt [89].

*miR-21* is induced during hypoxia by HIF-1 and leads to Akt activation [90]. On the other hand, *miR-26* is reduced up on oxygen deprivation [91], having potential inhibitory effect on Act activation during hypoxia. Interestingly, although *miR-221/222* expression was not reported to be affected by hypoxia, *miR221/222* overexpression in Dicer-knockdown endothelial cells restored the elevated eNOS protein levels that was induced by Dicer silencing [92].

Recent reports also provided evidence that *miR-486* is upregulated during hypoxia and contributes to VEGF signaling in bone marrow-derived mesynechymal stem cells [93].

HIF-1 drives Importantly, *miR-155* that targets mRNA of Src homology-2 domain-containing inositol 5 ∼ phosphatase 1 (SHIP1) as well [94]. SHIP1 is a PIP3 phosphatase that deactivates Akt [94]. Hence, *miR-155* impacts on NO levels during hypoxia may result from both regulation of *NOS3* expression and eNOS activity through Akt. It is possible that *miR-155* reduces *NOS3* mRNA levels, but at the same time prevents loss of NO via stimulating eNOS activity.

Furthermore, the phosphatidylinositol 3 kinase (PI3K) is the activator of the Akt pathway and is targeted by *miR-126* [95], the only miRNA considered to be specially expressed in endothelial cells and hematopoietic progenitor cells [96]. Hence, the observed hypoxic downregulation of *miR-126* contributes to increased VEGF expression and Akt activation [97].

It has to be emphasized here that hypoxia interactions via increased Akt-mediated serine phosphorylation during hypoxia disrupts hnRNP E1/*NOS3* 3′-UTR interactions, allowing *sONE*- and *miR-765*-dependent destabilization of *NOS3* mRNA [25]. Hence, as best illustrated by *miR-155*, further studies are required to understand the interplay between the negative effects of hypoxic Act activation on *NOS3* levels and the positive effect on eNOS activity.

During hypoxia, oxygen deprivation affects the levels ofthe soluble guanylyl cyclase (sGC) that is the principal receptor for NO, and thus provides another level of regulation of eNOS activity [98, 99]. Importantly, a recent study reported that hypoxia reduces the sGC levels in mice and decreases NO-stimulated sGC activity [100]. The same study that hypoxia induced murine *miR-34a-5p* to directly downregulate sGC expression, and thus prevent NO signaling [100]. However, whether *miR-34a-5p* has a similar impact on sGC in man requires further study.

Another interesting report identified an intronic “*27-nt miRNA*” derived from the 27-base pair repeats in intron 4 of *NOS3* gene [101]. “*27-nt miRNA*” overexpression indirectly downregulated *NOS3* mRNA and protein expression, and decreased the *NOS3* transcriptional efficiency [101]. However, this “*27-nt miRNA*” expression was not confirmed in human cells nor the specific promoter sequence for this potential miRNA was not identified. Hence, the further studies are required to evaluate the biological function of this miRNA candidate.

The majority of the studies conducted so far have only considered simplified models in which a single miRNA was analyzed in the context of one or more mRNA targets. Although these studies advance our understanding of how miRNAs function as cellular regulators, they neglect the fact that a single mRNA can be regulated by the simultaneous coordinated actions of a number of different miRNAs. For example, the *NOS3* 3′UTR is 429 base long, the *sONE* 3′UTR is 1717 base long), whereas the miRNA seed sequences are usually only 6-8 bases. Hence, *NOS3*/*sONE* RNAs can bind a combination of miRNAs simultaneously and these miRNAs will determine *NOS3* mRNA’s translation and stability. Furthermore, numerous reports conclude the biological role of miRNAs based on correlation studies only or limit their experiments to observing the effects of artificial miRNA overexpression. The latter approach ignores the importance of physiological steady-state levels of miRNAs and theseoften result in the identification of false positive targets.

Hypoxia provides an illustration of the bidirectional impact that miRNAs have on endothelial NO homeostasis. The fact that the very same miRNA simultaneously directly destabilizes *NOS3* mRNA and stimulates eNOS activity emphasizes the importance of maintaining the NO balance in both physiological and pathological conditions. This illustrates the need to understand miRNA’s role in eNOS biology and particularly with regard to the functional implications. Furthermore, the levels hypoxamiRs often change dynamically during hypoxia [49, 50], and thus the temporal effects need to be fully understood.

Interestingly, some hypoxamiRs like *miR-155* are also modulated by oxidative stress [102]. Hence, these miRNAs could provide an important sensor of eNOS uncoupling and function as a “safety switch”. However, validation of this hypothesis requires further study.

It is also worthwhile to note that the endothelium from different vascular beds varies in its molecular responses to hypoxia, including *NOS3* and *sONE* mRNAs expression, as well as the expression of the miRNAs. Also, the results are often conflicting regarding the changes in miRNA levels during the hypoxia time course [50, 103]. Hence, we speculate that endothelium-related miRNA expression in the different organs can be responsible for the specific eNOS-dependent NO signaling in response to hypoxia/ischemia.

## Concluding remarks

**Fig. 3:**
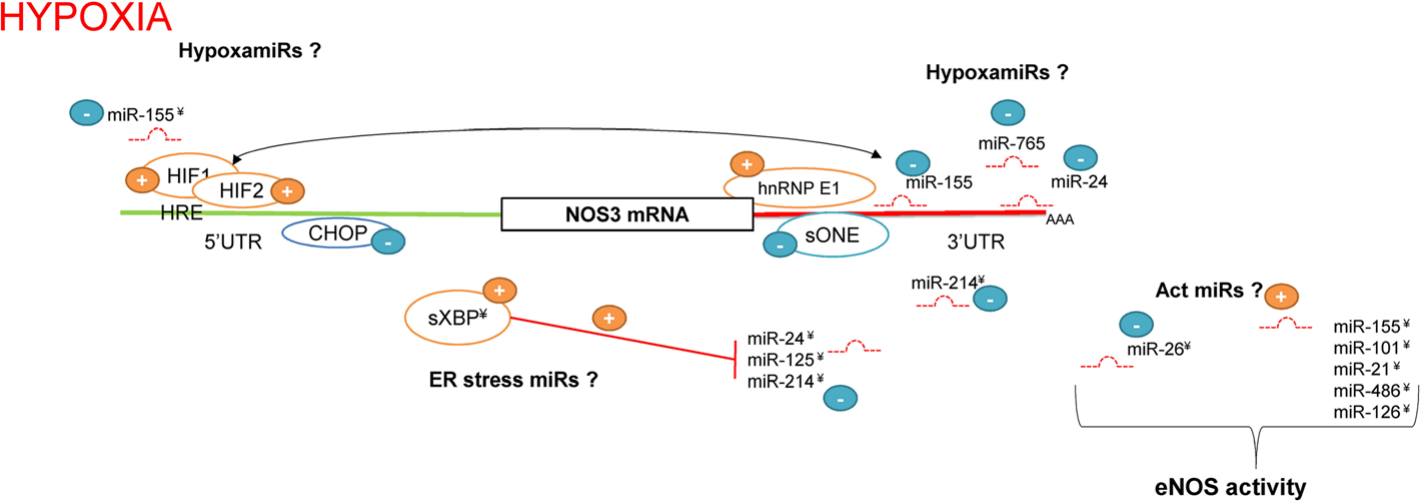
The transcriptional and posttranscriptional influence of hypoxia on *NOS3* mRNA levels. During hypoxia, HIF-1 and HIF-2 accumulate in the nucleus, where they bind to a sequence in the promoter region of *NOS3* termed the hypoxia-response element (HRE), and in doing so, induce *NOS3* expression. Hypoxia disrupts hnRNP E1/*NOS3* 3′-UTR interactions and makes this transcript susceptible to *sONE* and miRNA-related down-regulation. HIF-1 induces miR-155 that along with miR-765 and miR-24 destabilizes *NOS3* mRNA. Furthermore, during hypoxia, miR-214 has negative effect on *NOS3* expression. Hypoxia is accompanied by deregulation of ER homeostasis and HIF-1-related activation of the ER stress response. The proadaptive and proangiogenic ER stress transcription factor sXBP1 stimulates *NOS3* expression transcriptionally and postranscriptionally through reduction of miR-24, miR-125 and miR-214. However, another proapoptotic ER stress transcription factor, CHOP, binds to the 5′UTR and represses transcription. The hypoxamiRs also modulate Act signaling pathway and consequently eNOS activity. During hypoxia, the changes in the expression levels of miR-155, miR-101, miR-486, miR-21 and miR-126 stimulate Akt activation, whereas miR-26 prevents Act signaling. The (**+**) expression profile changed during hypoxia contributes to increased expression and activity of *NOS3*; the (**-**) expression profile changed during hypoxia has a negative effect on the expression and activity of *NOS3*; ^(¥)^ depicts indirect effects on the *NOS3* gene

It is clear that understanding the cellular pathways that regulate NO bioavailability in human endothelium under hypoxia is necessary in order to develop novel treatments for cardiovascular disorders. The endothelial *NOS3* expression and NO bioavailability during hypoxia results from a complicated equilibrium that exist between the proapoptotic and survival pathways as summarized in Fig. 3. Although the molecular mechanisms of the cellular response to hypoxia and ER stress have been extensively studied, there is limited information regarding their interplay and the role of miRNAs in these processes. Furthermore, a large number of both transcriptional and posttranscriptional factors that maintain endothelial NO homeostasis [104] can also be involved in the miRNAs regulatory networks.

Considering the recent interest in the development of novel micro-RNA-based therapies for human pathologies, it is tempting to speculate that miRNAs or their analogs offer a novel therapeutic approach in regulating endothelial NO bioavailability. Although we can modulate the cellular miRNA levels (either with their analogs (miRNA overexpression) or inhibitors (miRNA reduction), the multiple target genes of miRNAs remain a main limitation of such strategies. Since a single miRNA can regulate hundreds of different mRNAs, an alteration in this miRNA expression pattern will have a wide range of consequences for cell metabolism. *miR-155* provides a perfect example how wide and complex potential influence of single miRNA is on eNOS activity. Understanding the miRNA governed balance between decreasing *NOS3* expression and increasing eNOS activity is crucial for the development of further therapeutic approaches.

Fortunately, a novel alternative approach relies on target protectors that add specificity to the mRNA of interest. Target protectors are single-stranded, modified RNAs that inhibit the interaction of the miRNA with a specific target without blocking the effects of the particular miRNA on other targets [105]. Hence, target protectors might appear to be extremely helpful tools in sorting out complex role of miRNA in maintaining NO homeostasis. And finally, one has to be aware of the cell- and tissue-specific differences in miRNA expression during hypoxia. Despite these concerns, understanding the role of miRNAs in modulating NO bioavailability will remain an active area of research.

## Abbreviations

3′- UTR: 3′-untranslated region
eNOS: NOS3 - endothelial nitric oxide synthase
HIF: Hypoxia-inducible factor
hnRNP L: Heterogeneous nuclear ribonucleoprotein L
HRE: Hypoxia-response element
miRNA: microRNA
NO: Nitric oxide
O_2_^-^: Superpoxide
ONOO^-^: Peroxynitrite
RISC: miRNA-induced silencing complex
VEGF: Vascular endothelial growth factor
VEGFR2: Vascular endothelial growth factor receptor-2
VHL: Gene encoding von Hippel-Lindau tumor suppressor protein
